# Supplementary material for: Detection of BRAF mutations in malignant melanoma and colorectal cancer by SensiScreen® FFPE BRAF qPCR assay
Source: PLoS One. 2023 Feb 9;18(2):e0281558. doi: 10.1371/journal.pone.0281558 (PMC9910728; doi:10.1371/journal.pone.0281558)
Supplement: S1 Fig — (DOCX) [file pone.0281558.s001.docx]

**Supplementary Figure 1.** Calibration curves of SensiScreen^®^ FFPE BRAF qPCR Assay for each target Ct value as function of log (copy number).

**Simplex**

**Multiplex**
