# Supplementary material for: Detection of BRAF mutations in malignant melanoma and colorectal cancer by SensiScreen® FFPE BRAF qPCR assay
Source: PLoS One. 2023 Feb 9;18(2):e0281558. doi: 10.1371/journal.pone.0281558 (PMC9910728; doi:10.1371/journal.pone.0281558)
Supplement: S1 Table — Abbreviations; F: female; M: male. (DOCX) [file pone.0281558.s002.docx]

**Supplementary Table 1: Clinical-pathological characteristics of cohort 1 and 2 used for analysis by Cobas^®^ 4800, ME-PCR and DS.** Abbreviations: F, female; M, male.

| **Cohort 1 (malignant melanoma)** | | | | **Cohort 2 (colorectal cancers)** | | | |
| --- | --- | --- | --- | --- | --- | --- | --- |
| **n** | **Sex** | **Age** | **Localization** | **n** | **Sex** | **Age** | **Localization** |
| 1 | F | 65 | lymph node, inguen | 1 | M | 56 | sigmoid colon |
| 2 | M | 43 | cerebrum | 2 | M | 80 | sigmoid colon |
| 3 | F | 50 | cavum nasi (sinus) | 3 | F | 69 | sigmoid colon |
| 4 | F | 78 | urethra | 4 | M | 67 | sigmoid colon |
| 5 | M | 29 | subcutis, shoulder | 5 | M | 93 | sigmoid colon |
| 6 | M | 82 | axcil | 6 | F | 78 | rectum |
| 7 | F | 67 | subcutis, truncus | 7 | M | 56 | cecum |
| 8 | M | 81 | back | 8 | M | 74 | cecum |
| 9 | M | 76 | skin, thigh | 9 | M | 61 | sigmoid colon |
| 10 | M | 64 | lymph node, inguen | 10 | M | 71 | sigmoid colon |
| 11 | M | 83 | liver | 11 | M | 69 | hepatic flexure |
| 12 | F | 63 | subcutis, truncus | 12 | F | 91 | cecum |
| 13 | F | 65 | forehead | 13 | M | 75 | sigmoid colon |
| 14 | M | 75 | lower back | 14 | M | 83 | transverse colon |
| 15 | M | 73 | liver | 15 | M | 76 | ascending colon/cecum |
| 16 | M | 72 | forearm | 16 | F | 81 | cecum |
| 17 | M | 73 | cerebrum | 17 | F | 75 | cecum |
| 18 | F | 44 | retroperitoneum | 18 | M | 71 | descending colon/cecum |
| 19 | M | 71 | liver | 19 | F | 71 | hepatic flexure |
| 20 | M | 64 | mediastinum | 20 | F | 75 | sigmoid colon |
| 21 | F | 80 | jejenum | 21 | M | 84 | sigmoid colon |
| 22 | F | 63 | paranasal sinus, basal cranii | 22 | M | 79 | ascending colon/cecum |
| 23 | M | 76 | subcutis | 23 | M | 71 | sigmoid colon |
| 24 | M | 75 | lymph node, neck | 24 | M | 73 | rectum |
| 25 | F | 85 | abdomen | 25 | M | 61 | sigmoid colon |
| 26 | F | 64 | subcutis, leg | 26 | F | 90 | rectum |
| 27 | F | 85 | lymph node, inguen | 27 | M | 80 | cecum |
| 28 | M | 55 | lymph node, neck | 28 | F | 91 | sigmoid colon |
| 29 | M | 70 | lymph node, axil | 29 | F | 83 | sigmoid colon |
| 30 | K | 70 | lung | 30 | F | 81 | sigmoid colon / rectum |
| 31 | F | 56 | lung | 31 | M | 62 | sigmoid colon |
| 32 | M | 67 | cerebrum | 32 | M | 81 | rectum |
| 33 | F | 70 | axil | 33 | F | 96 | sigmoid colon |
| 34 | M | 79 | forearm | 34 | F | 59 | transverse colon |
| 35 | M | 48 | sentinel node | 35 | M | 76 | ascending colon/cecum |
| 36 | M | 57 | cerebrum | 36 | M | 77 | cecum |
| 37 | M | 60 | subcutis | 37 | F | 73 | ascending colon |
| 38 | M | 80 | lymph node, neck | 38 | F | 73 | sigmoid colon |
| 39 | M | 75 | lymph node | 39 | F | 81 | ascending colon |
| 40 | M | 84 | glandula parotidea | 40 | F | 46 | ascending colon |
| 41 | M | 48 | back | 41 | M | 79 | descending colon |
| 42 | M | 60 | face | 42 | F | 80 | hepatic flexure |
| 43 | F | 70 | cerebrum | 43 | F | 70 | ascending colon |
| 44 | M | 34 | lymph node, pelvis | 44 | F | 66 | descending colon |
| 45 | F | 40 | lung | 45 | F | 64 | cecum |
| 46 | M | 55 | soft tissue, lower back | 46 | F | 69 | transverse colon |
| 47 | F | 55 | liver | 47 | M | 84 | rectum |
| 48 | M | 71 | dura mater | 48 | M | 49 | ascending colon |
| 49 | M | 75 | cerebrum | 49 | M | 60 | sigmoid colon |
| 50 | M | 73 | ventricle, mucosa | 50 | M | 71 | rectum |
| 51 | F | 74 | lower leg | 51 | M | 64 | ascending colon |
| 52 | M | 77 | lymph node, inguen | 52 | F | 70 | ascending colon |
| 53 | M | 50 | cerebrum | 53 | M | 70 | ascending colon |
| 54 | M | 54 | lymph node, truncus | 54 | F | 65 | sigmoid colon |
| 55 | F | 76 | lymph node, inguen | 55 | F | 77 | ascending colon |
| 56 | F | 77 | lymph node, axil | 56 | F | 81 | sigmoid colon |
| 57 | M | 47 | lymph node, inguen | 57 | F | 72 | sigmoid colon |
| 58 | M | 71 | lymph node, neck | 58 | M | 83 | ascending colon |
| 59 | M | 41 | lymph node, inguen | 59 | F | 87 | descending colon |
| 60 | M | 85 | shoulder | 60 | M | 77 | rectum |
| 61 | M | 65 | lung | 61 | F | 80 | cecum |
| 62 | F | 85 | toe | 62 | M | 76 | hepatic flexure |
| 63 | F | 69 | right lung | 63 | M | 78 | cecum |
| 64 | M | 80 | temple | 64 | M | 77 | sigmoid colon |
| 65 | F | 71 | back | 65 | M | 76 | splenic flexure |
| 66 | F | 42 | lymph node, inguen | 66 | F | 59 | descending colon |
| 67 | M | 63 | lymph node, axil | 67 | M | 61 | ascending colon |
| 68 | M | 86 | axil | 68 | M | 88 | sigmoid colon |
| 69 | F | 49 | thigh | 69 | F | 78 | ascending colon |
| 70 | M | 66 | lymph node,neck | 70 | M | 47 | hepatic flexure |
| 71 | M | 41 | back | 71 | F | 78 | cecum |
| 72 | M | 70 | cerebrum | 72 | F | 60 | ascending colon |
| 73 | F | 71 | truncus | 73 | M | 50 | sigmoid colon / rectum |
| 74 | F | 35 | back | 74 | F | 71 | cecum |
| 75 | M | 80 | foot | 75 | M | 74 | hepatic flexure |
| 76 | M | 79 | axil | 76 | F | 93 | cecum |
| 77 | F | 61 | forearm | 77 | M | 65 | transverse colon |
| 78 | M | 75 | upper arm | 78 | F | 90 | cecum |
| 79 | F | 69 | lower back | 79 | M | 60 | sigmoid colon |
| 80 | M | 82 | mammae | 80 | M | 77 | cecum |
| 81 | M | 76 | back | 81 | M | 78 | cecum |
| 82 | F | 86 | lymph node, inguen | 82 | F | 78 | sigmoid colon |
| 83 | M | 44 | cerebellum | 83 | M | 42 | descending colon |
| 84 | F | 67 | lymph node, axil | 84 | F | 65 | hepatic flexure |
| 85 | M | 66 | ear | 85 | F | 62 | sigmoid colon / rectum |
| 86 | M | 85 | ear | 86 | F | 61 | cecum |
| 87 | M | 72 | shoulder | 87 | M | 85 | hepatic flexure |
| 88 | M | 72 | lymph node, neck | 88 | F | 78 | descending colon |
| 89 | F | 41 | soft tissue, inguen | 89 | F | 87 | descending colon |
| 90 | F | 79 | back | 90 | F | 80 | sigmoid colon |
| 91 | F | 56 | lymph node, inguen | 91 | F | 84 | cecum |
| 92 | F | 68 | vulva | 92 | M | 73 | descending colon |
| 93 | F | 83 | lower leg | 93 | F | 54 | ascending colon |
| 94 | M | 75 | truncus | 94 | M | 82 | hepatic flexure |
| 95 | F | 74 | liver | 95 | M | 64 | cecum |
| 96 | F | 41 | leg | 96 | M | 88 | splenic flexure |
| 97 | M | 41 | colon descendens | 97 | F | 69 | transverse colon |
| 98 | M | 72 | back | 98 | F | 85 | cecum |
| 99 | M | 69 | knee | 99 | F | 85 | ascending colon |
| 100 | M | 60 | lymph node | 100 | F | 72 | rectum |
| 101 | M | 78 | breast / thorax |  |  |  |  |
| 102 | M | 55 | lymph node, inguen |  |  |  |  |
| 103 | F | 51 | vulva |  |  |  |  |
| 104 | F | 48 | lymph node, inguen |  |  |  |  |
| 105 | F | 56 | lung |  |  |  |  |
| 106 | M | 66 | skin, flank |  |  |  |  |
| 107 | M | 78 | breast / thorax |  |  |  |  |
| 108 | M | 64 | truncus |  |  |  |  |
| 109 | M | 77 | lymph node |  |  |  |  |
| 110 | M | 66 | face |  |  |  |  |
| 111 | M | 45 | face |  |  |  |  |
| 112 | F | 75 | lymph node, abdomen |  |  |  |  |
| 113 | M | 69 | thigh |  |  |  |  |
| 114 | M | 65 | neck |  |  |  |  |
| 115 | F | 56 | upper arm |  |  |  |  |
| 116 | F | 83 | knee |  |  |  |  |
| 117 | F | 38 | back |  |  |  |  |
| 118 | F | 74 | lymph node, neck |  |  |  |  |
| 119 | M | 66 | subcutis |  |  |  |  |
| 120 | M | 63 | nervus opticus |  |  |  |  |
| 121 | M | 51 | NA |  |  |  |  |
| 122 | M | 79 | lymph node, axil |  |  |  |  |
| 123 | F | 72 | lower leg |  |  |  |  |
