# Supplementary material for: Detection of BRAF mutations in malignant melanoma and colorectal cancer by SensiScreen® FFPE BRAF qPCR assay
Source: PLoS One. 2023 Feb 9;18(2):e0281558. doi: 10.1371/journal.pone.0281558 (PMC9910728; doi:10.1371/journal.pone.0281558)
Supplement: Attachment — Submitted filename: Response to Reviewers.docx [file pone.0281558.s003.docx]

December 2022

Dear Editor Alvaro Galli and reviewers

We thank you for the careful and constructive review of our manuscript, and for the willingness to let us submit a revision of the manuscript for review in PLOS ONE. The comments have been very helpful and have greatly improved the manuscript.

We have addressed the comments and the manuscript has been revised accordingly. Please find the reviewer comments and our responses below.

Best regards

Anna Lahn Sørensen, PhD

**Journal Requirements:**

When submitting your revision, we need you to address these additional requirements.

1. Please ensure that your manuscript meets PLOS ONE's style requirements, including those for file naming. The PLOS ONE style templates can be found at 
<https://journals.plos.org/plosone/s/file?id=wjVg/PLOSOne_formatting_sample_main_body.pdf> and 
<https://journals.plos.org/plosone/s/file?id=ba62/PLOSOne_formatting_sample_title_authors_affiliations.pdf>.

Fig 1.tiff was renamed to Fig1.tiff

2. Please provide additional details regarding participant consent. In the ethics statement in the Methods and online submission information, please ensure that you have specified (1) whether consent was informed and (2) what type you obtained (for instance, written or verbal, and if verbal, how it was documented and witnessed). If your study included minors, state whether you obtained consent from parents or guardians. If the need for consent was waived by the ethics committee, please include this information.

At the time of diagnosis all the patients included in the study gave a verbal general consent permitting the use of the tissue material for each kind of research. This information was written by the clinicians in the medical report. Only patients >18 years old were enrolled in the study. We have not a written informed consent because our cases were diagnosed before 2010 and were analyzed in a period from 2010 to 2012. At that time, there was not the need to obtain a written consent for doing this kind of research on tissue from histological archives.

If you are reporting a retrospective study of medical records or archived samples, please ensure that you have discussed whether all data were fully anonymized before you accessed them and/or whether the IRB or ethics committee waived the requirement for informed consent. If patients provided informed written consent to have data from their medical records used in research, please include this information.

This has been corrected (line 191).

3. Thank you for stating the following in the Competing Interests section:
"ALS, MB, RKP, EPO, KF, MHK, JL and UBC received salaries from Eurostars. ALS, MB, RKP, EPO, KF and UBC are employees of PentaBase A/S. SensiScreen FFPE BRAF qPCR is part of a marketed product portfolio of PentaBase A/S."

We note that one or more of the authors have an affiliation to the commercial funders of this research study : PentaBase A/S.

a) Please provide an amended Funding Statement declaring this commercial affiliation, as well as a statement regarding the Role of Funders in your study. If the funding organization did not play a role in the study design, data collection and analysis, decision to publish, or preparation of the manuscript and only provided financial support in the form of authors' salaries and/or research materials, please review your statements relating to the author contributions, and ensure you have specifically and accurately indicated the role(s) that these authors had in your study. You can update author roles in the Author Contributions section of the online submission form.

This has been updated in the cover letter

Please also include the following statement within your amended Funding Statement. 
“The funder provided support in the form of salaries for authors [insert relevant initials], but did not have any additional role in the study design, data collection and analysis, decision to publish, or preparation of the manuscript. The specific roles of these authors are articulated in the ‘author contributions’ section.”
If your commercial affiliation did play a role in your study, please state and explain this role within your updated Funding Statement.

This has been updated in the cover letter

b) Please also provide an updated Competing Interests Statement declaring this commercial affiliation along with any other relevant declarations relating to employment, consultancy, patents, products in development, or marketed products, etc.

This has been updated in the cover letter

Within your Competing Interests Statement, please confirm that this commercial affiliation does not alter your adherence to all PLOS ONE policies on sharing data and materials by including the following statement: "This does not alter our adherence to  PLOS ONE policies on sharing data and materials.” (as detailed online in our guide for authors <http://journals.plos.org/plosone/s/competing-interests>). If this adherence statement is not accurate and  there are restrictions on sharing of data and/or materials, please state these. Please note that we cannot proceed with consideration of your article until this information has been declared.

This has been updated in the cover letter

Please include both an updated Funding Statement and Competing Interests Statement in your cover letter. We will change the online submission form on your behalf.

This has been updated in the cover letter.

**Review Comments to the Author**

Reviewer #1: In this study, Authors presented a new methodology based on a quantitative PCR assay for detection of BRAF mutation in melanoma and colorectal cancer. The SensiScreen® FFPE BRAF qPCR Assay here proposed is able to identify not only the V600E variant of BRAF, but also other clinically-relevant mutations in such a gene.
The experimental plan is well developed and description of data is detailed.
My only criticism is the following: it is not appropriate to affirm - into the Discussion section - that the SensiScreen FFPE BRAF qPCR Assay may represent "a better option than NGS, a method characterized by lower sensitivity (5% in fixed tissues) and a longer procedure at least two working days compared to less than 2 hours for the SensiScreen® FFPE BRAF qPCR Assay". The qPCR and NGS approaches ARE TWO DISTINCT MOLECULAR DIAGNOSTIC FIELDS! The comparison of the assay here proposed should be with the other existing quantitative PCR assays (as correctly done into the work). Therefore, this sentence must be deleted since it is misleading.

We agree, and the sentence has been removed.

Reviewer #2: The present study brings another PCR based approach for BRAF mutation detection. It does lack novelty, in a very active market, with point-of care, nanosolutions, etc, and the new methodology is compared with old fashion approaches, such as direct sequencing, which are no longer use in the routine setting.

We agree that today direct sequencing is not used extensively for diagnosis. The direct sequencing data has been a starting point to evaluate how many patients were mutant or not in our cohort and was a common methodology at the time in which we implemented the assay reported in this paper. To limit the problem of direct sequencing low sensitivity we have also done Mutant-enriched PCR which has been compared to SensiScreen® FFPE BRAF qPCR Assay.

Extra issues that should be addressed by the authors:

1- Currently the methodologies available with higher sensitive are NGS and ddPCR. Since the aim of the study, as mentioned by the authors, is to “was to validate a highly sensitive, specific, fast, and easy-to-use assay for BRAF”. Most of the advantageous can be found in both NGS and ddPCR. Importantly, It is not anymore acceptable to run against direct sequencing, which has been known for the high LOD rate, and since almost a decade years is not the gold standard for mutation detection. So, the comparison made in cohort 2, is meaningless.

We agree with you that detection of BRAF mutation is occurring more commonly with NGS or ddPCR nowadays. Due to the lacking guidelines of specific method for detecting of BRAF mutation we have chosen to compare our SensiScreen BRAF qPCR with direct sequencing, ME-PCR and Cobas BRAF mutation assay. We have addressed this in the manuscript’s discussion (line 357-362).

2- The introduction most explain the readers the methodology and underlying basis of the PentaBase DNA analogue technology, the core methodology of the manuscript, instead of repletion of BRAF clinical impact that is very long in the introduction and can be synthetize.

We agree, we have removed BRAF clinical impact in the introduction and instead added the underlying basis of PentaBase DNA analogue technology (line 147-150).

In the material and methods:
3-LOB, was not done?

It was done and it has been included in the manuscript (line 177-178, 300-302 and table 3).

4- “Only patients with sufficient tumour tissue material have been include. Be more precise and rigorous genomic DNA was extracted from six 7 μm thick serial FFPE sections” – this is too much for any current methodology and does not meet the goal of the authors of “SensiScreen® FFPE BRAF qPCR Assay was developed to use a minimal input of DNA.”

We agree, it is too much. DNA was extracted from six 7 μm thick serial, and only a small amount of DNA was used for the assay. We have added a line stating that only five µL of 1-10 ng/ µL DNA is necessary (line 265-268). We hope this clarify the misleading text.

5- Clarify - Four patients (cohort 1) and three patients (cohort 2) were excluded due to invalid DNA for the present assay only ? So, the Roche assay and DS had reliable results? How the new assay con overperform the COBAS and DS in these cases?

We now see that this can be misleading. We have tried to clarify it in the manuscript. In cohort 1 the four patients were removed from the tests, due to insufficient DNA. The purified DNA was not sufficient to run either the Cobas or SensiScreen. To clarify line 211 has been modified. In cohort 2 three patients were excluded due to the quality of the DNA. The patient samples had initially been identified with direct sequencing. Long term storage resulted in insufficient and invalid DNA, the three patients were due to this, excluded from the SensiScreen test. To clarify line 217-218 have been modified.

6- Spell out ME-PCR in MM

It has been spelled out in the manuscript.

7- Discussion should address the price of assays and cost-effective of the methodologies.

It has been included in the manuscript (line 383-386).
